# Onychotillomania in the Setting of Homelessness

**DOI:** 10.7759/cureus.22988

**Published:** 2022-03-09

**Authors:** Taha F Rasul, Sana Gulraiz, Armen Henderson

**Affiliations:** 1 Department of Infectious Diseases, University of Miami Miller School of Medicine, Miami, USA; 2 School of Public Health, West Virginia University School of Medicine, Morgantown, USA; 3 Internal Medicine, University of Miami Hospital, Miami, USA

**Keywords:** multidisciplinary teams, street medicine, psychodermatosis, nail diseases, skin picking, homeless persons, onychotillomania

## Abstract

Onychotillomania is a psychodermatosis that involves repetitive, self-induced trauma to the nail and sometimes the periungual skin. It is generally seen as an overlapping psychiatric and dermatologic disorder, although there have not been any statistically significant associations with psychiatric illness. Some studies have noted an association with obsessive-compulsive disorder (OCD). Due to the relative lack of empirical data on this condition, treatments are often not evidence-based. As a result, there is no standardized method of treating onychotillomania, and patients suffering from this disease are susceptible to relapse. This report presents the case of a 32-year-old male experiencing homelessness and suffering from major depressive disorder and methamphetamine use disorder who developed onychotillomania two months after becoming homeless. He regularly used various instruments such as nail cutters, tweezers, and nail files to constantly pick at his nails, a few of which were noted to be bleeding with signs of infection. He was evaluated jointly by dermatology and psychiatry providers who confirmed the diagnosis. By thorough examination of the patient’s history, he was provided tactile sensory equipment to reduce his repetitive picking behavior. A direct referral for substance use counseling was also provided. At follow-up, he was noted to have a subjective improvement in his picking symptoms, although there was no significant difference in the size of his nails. This case represents the twofold challenge of managing a difficult condition, onychotillomania, in the setting of the severe socio-personal stressor of homelessness.

## Introduction

Onychotillomania is the self-induced trauma to the nail unit, either through picking or pulling at the nails and with occasional use of instruments [1]. It affects 0.9% of the population and has been noted to have an association with psychiatric diagnoses such as persistent depressive disorder and hypochondriasis. It is important to differentiate this condition from onychophagia, whereby the nails are chewed or bitten, or dermatillomania, where the skin surrounding the nail (or on other body parts) is picked or bitten [2]. The Diagnostic and Statistical Manual-5 (DSM-V) indirectly classifies onychotillomania as a body-focused repetitive behavior and further categorized it as an excoriation disorder. The intertwined psychiatric and cutaneous relationship is why it is often called a psychodermatosis [3]. In certain cases, onychotillomania has also been categorized among obsessive-compulsive disorders (OCD). This condition may lead to irreversible and severe nail dystrophy, infections (paronychia), or melanonychia [4].

No large clinical trials have currently been conducted to assess the efficacy of treatments. This may be in part due to the rarity of the condition. However, cognitive-behavioral therapy, physical barrier methods, and pharmacologic treatments have shown some benefits in certain cases. As a result, there are no evidence-based guidelines or treatment methods, which is why onychotillomania remains a clinical challenge to the multidisciplinary specialists involved in care.

It has been well established that homeless individuals have dramatically worse healthcare outcomes, as well as decreased quality and quantity of life, compared to the general population [5]. Certain estimates predict that the average life expectancy of a homeless individual is 55 years, more than 20 years below the United States national average [6]. This is due to a combination of severe chronic disease, mental health issues, and drug overdoses. Therefore, homeless patients have a high likelihood of having a comorbid mental illness, which may predispose them toward the development of excoriation disorders.

Onychotillomania has not been well described in the literature among homeless individuals. In fact, there have not been any recorded case reports of homeless patients with this high-morbidity disorder [7]. The overall data on the cutaneous conditions of homeless patients has also been lacking: isolated shelters only in Massachusetts and California have reported various skin conditions among patients encountered. No other major studies have been conducted in major metropolitan areas in the United States, particularly in the South Florida region, which contains a large homeless population [8].

The diverse population and lack of funding for mental health facilities, particularly among the unsheltered homeless, mean that there are high numbers of individuals with untreated and undiagnosed mental health issues.

From an economics perspective, the coronavirus pandemic had the unexpected effect of raising the cost of living to levels unsustainable for many lower socioeconomic status residents. From late 2021 onward, it has been estimated that Miami-Dade County has become one of the most expensive regions to live in in the United States [9]. As a result of expired eviction moratoriums and a generally unsustainable cost of living, the issue of homelessness in the area has worsened considerably. Such factors would no doubt also worsen mental health. By some estimates, financial hardships have an overall detrimental effect on mental health and increase the likelihood of developing depression and anxiety [10]. Certain homeless outreach programs such as Miami Street Medicine often participate in the care of such patients. We present a case of a 32-year-old unsheltered homeless patient with onychotillomania likely exacerbated by financial and personal stressors.

## Case presentation

During a routine medical outreach clinic for unsheltered homeless patients, a 32-year-old well-nourished male was encountered with a chief concern of being excessively stressed. He described how he became homeless a few months ago, mostly due to being unable to afford the increased cost of living. His rent had increased the year prior, and he was unable to make timely payments. His initial job did not pay enough to cover all his expenses, so he began also working smaller, supplementary jobs. His maximum level of education attained was the completion of high school. He was able to stay afloat until the death of a family member, whereby he described falling into a deep depression for a week. As a result, he was terminated from his employment and had worsening mental health. He began to use methamphetamine as a means to cope with his situation, before eventually being evicted from his home for failure to pay rent.

He approached the medical outreach team while picking at his fingernails and maintaining eye contact. When asked about the damage to his fingernails, he described this as a coping mechanism that he could not voluntarily stop. The picking behavior was aimed not only at the fingernails but also at the toenails of both feet. He did not exhibit either picking behavior prior to becoming homeless.

**Figure 1:**
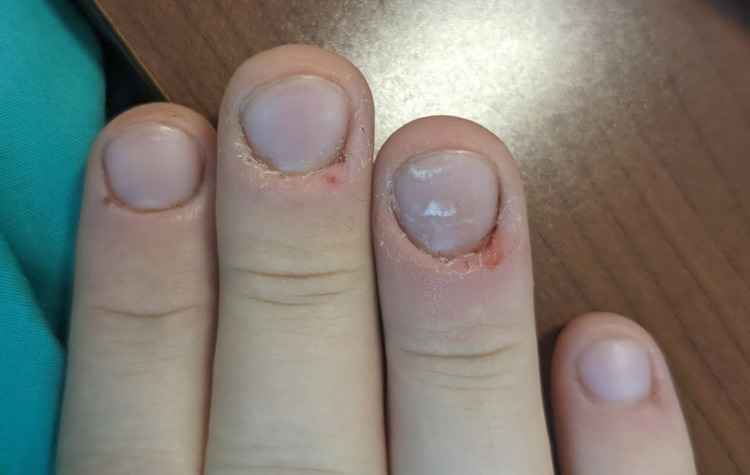
Initial photo of the right hand. There is a demonstrable partial loss of nails with trauma to the periungual areas, particularly on the fourth digit.

**Figure 2:**
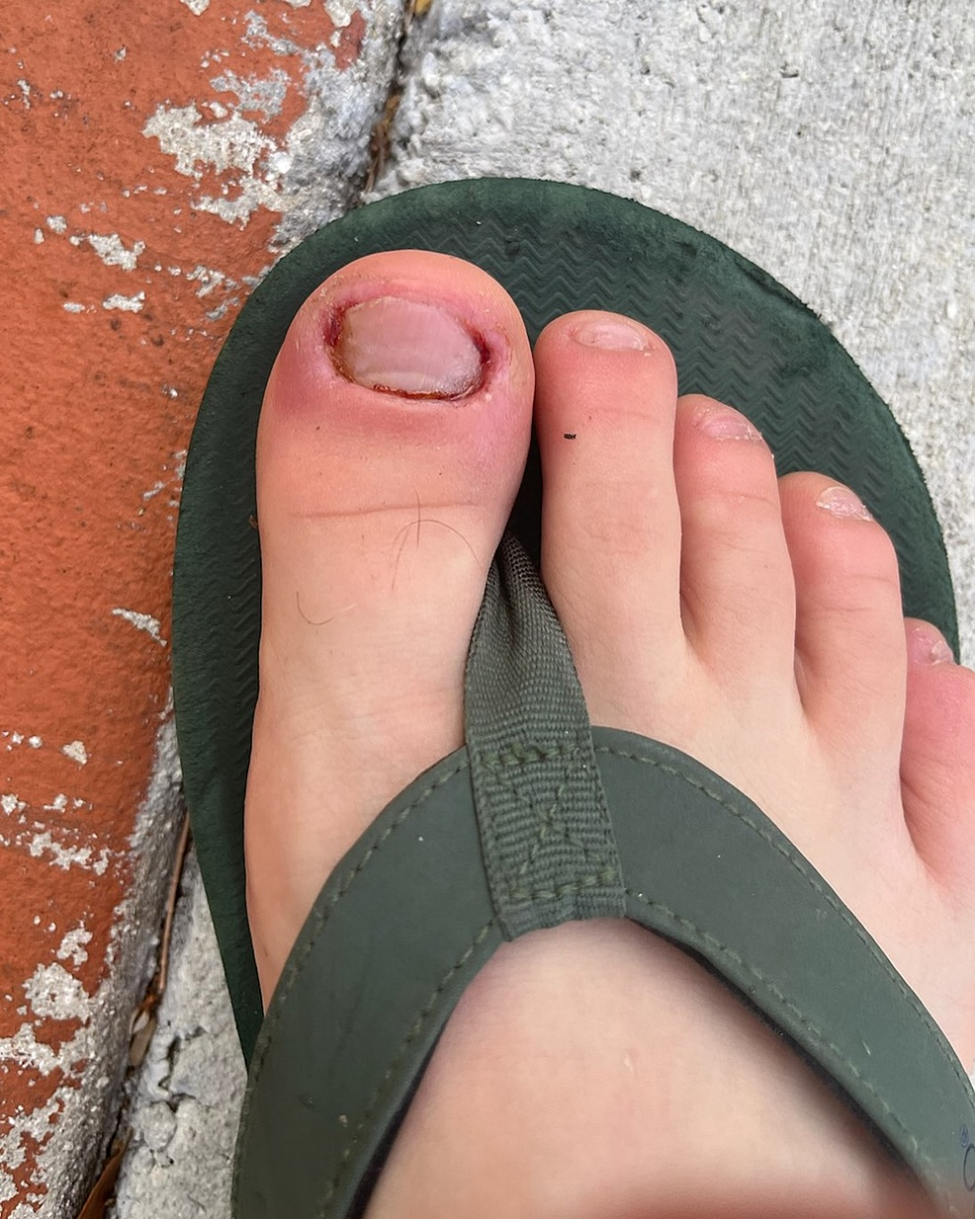
Initial survey of the right foot. Acute paronychia was noted on the hallux. All nails were significantly shortened with some dystrophic changes noted on the fifth toenail.

Upon initial survey, his right hand was noted to have nail shortening on all digits (Figure 1), and the patient endorsed having frequent infections around the nail beds of most of his fingers. In those cases, he described the pain as so severe that the use of his hands and feet would be limited. He later endorsed regular usage of methamphetamine. The patient reported continued onychotillomania during periods of sobriety, but when using, the behaviors worsened to also pick at his face, arms, and upper back. His right foot was also noted to have considerable damage and paronychia (Figure 2).

**Figure 3:**
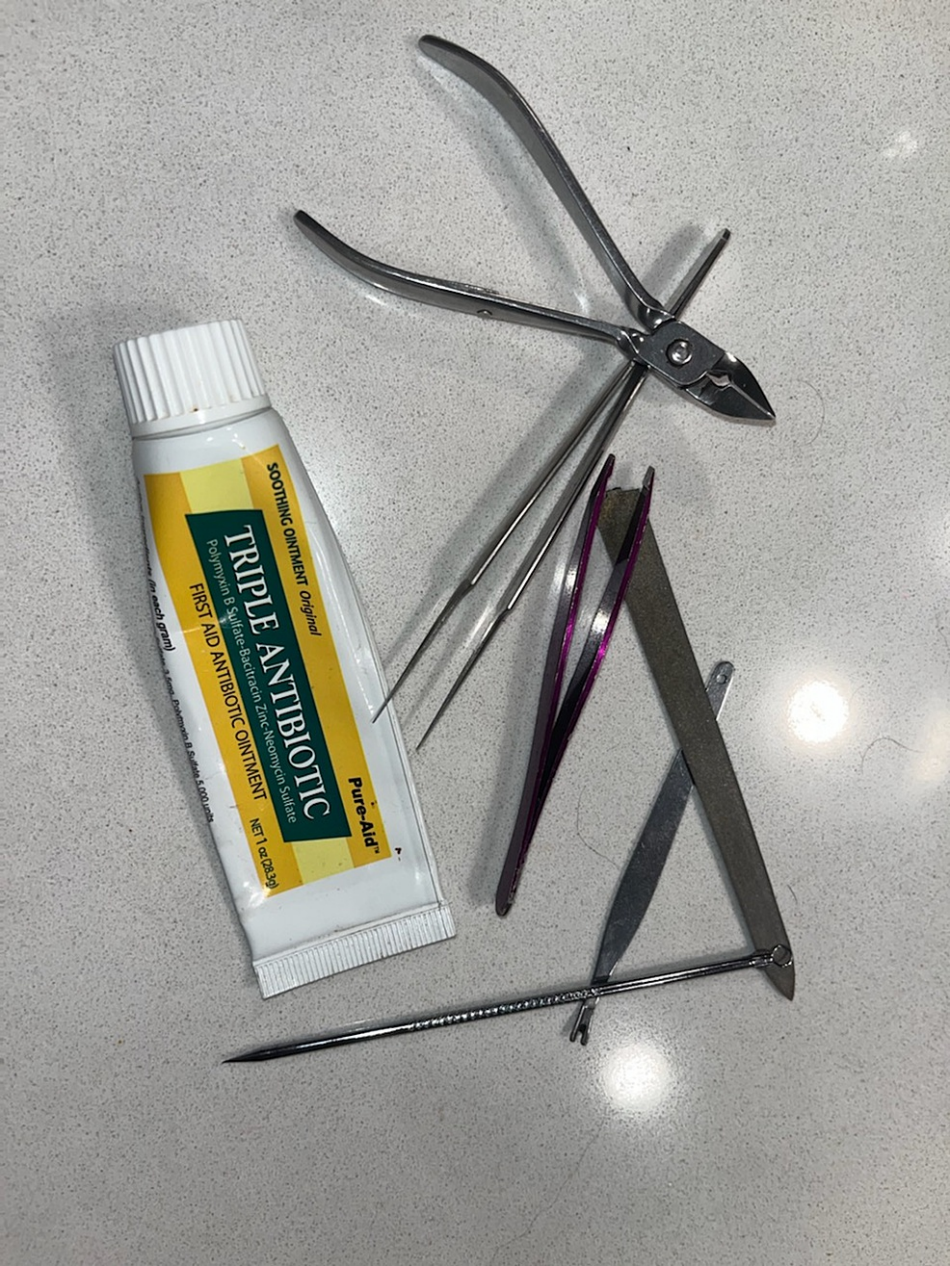
Tools and instruments used to damage the nails and surrounding skin. The antibiotic ointment would be used on actively bleeding areas.

His excoriation behavior was described by him as one of the many things in his life that he could not control. He also endorsed feeling a need to achieve perfection around the nail edges, thereby continuously “shaping” and altering the nail. Picking was not the only mechanism used; he collected multiple tools such as a nail file, tweezers, and a nail cutter in order to pick, pull, and cut the nails. He also kept topical antibiotic ointment (polymyxin, bacitracin, and neomycin) and applied it to the instruments and his fingers after causing excessive skin trauma (Figure 3).

After psychiatric and dermatology evaluation, it was determined that his condition was likely brought about by an adjustment disorder (or more chronic mood disorders such as major depressive disorder) compounded by active substance use. The street team provided him with counseling and distraction tools to avoid nail-picking. These included fidgeting toys (such as fidget spinners), moldable clay, and handheld cubes with different tactile surfaces. He was also referred to resources for the treatment of his substance use disorder.

**Figure 4:**
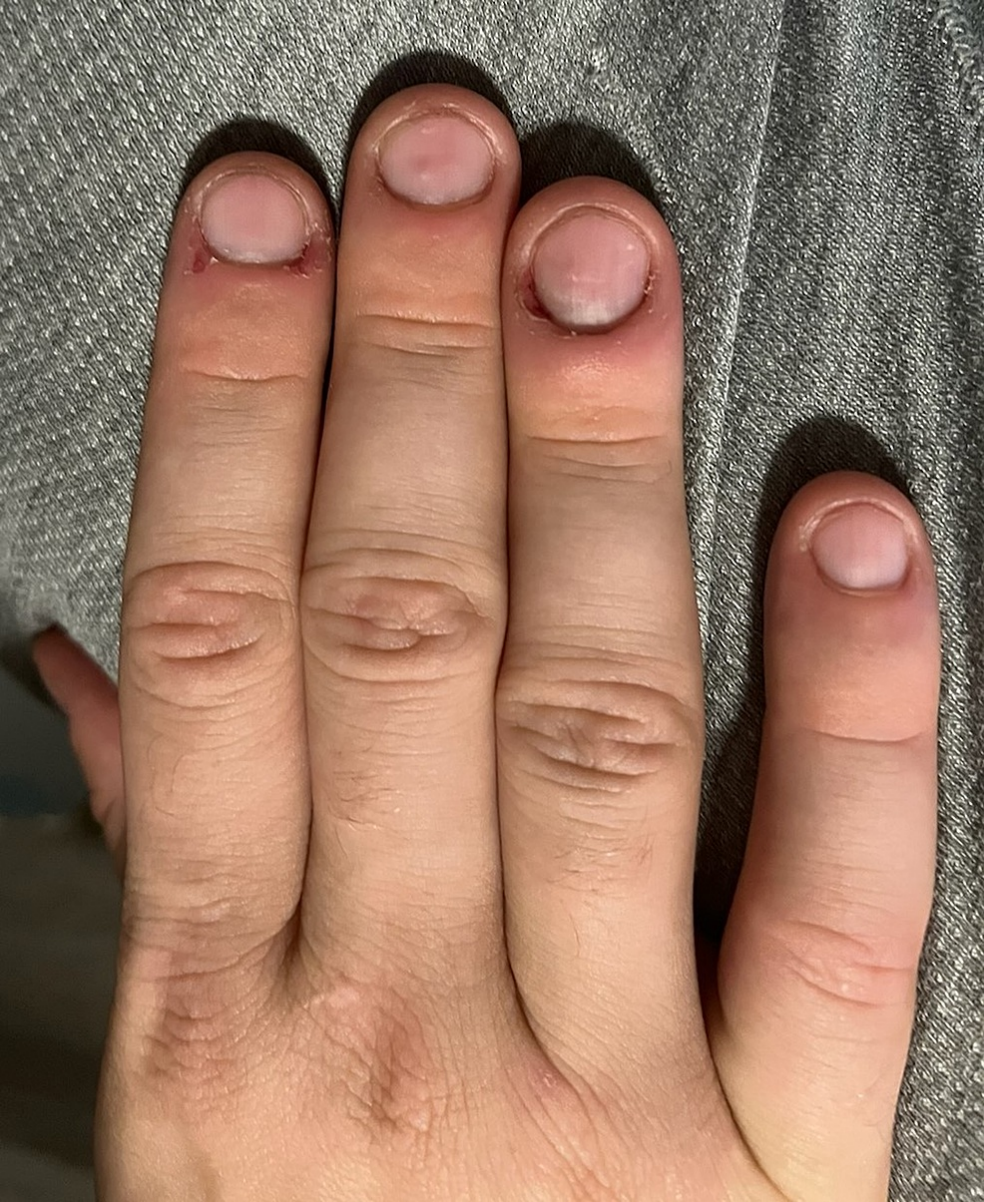
Four-week follow-up of the right hand. A slight improvement was noted in the periungual skin on the third and fourth digits, but no significant changes overall.

Four weeks after the initial visit, he was encountered again during a medical outreach clinic. He reported subjective improvement in his symptoms of compulsive picking. Although he was still using methamphetamine, he reported using decreased amounts compared to before. Additionally, during the interview, he was able to maintain eye contact appropriately and would occasionally pick at his fingers or use the molding clay provided previously. The nail beds, periungual skin, and nails on the hand did not have any noticeable improvement (Figure 4).

**Figure 5:**
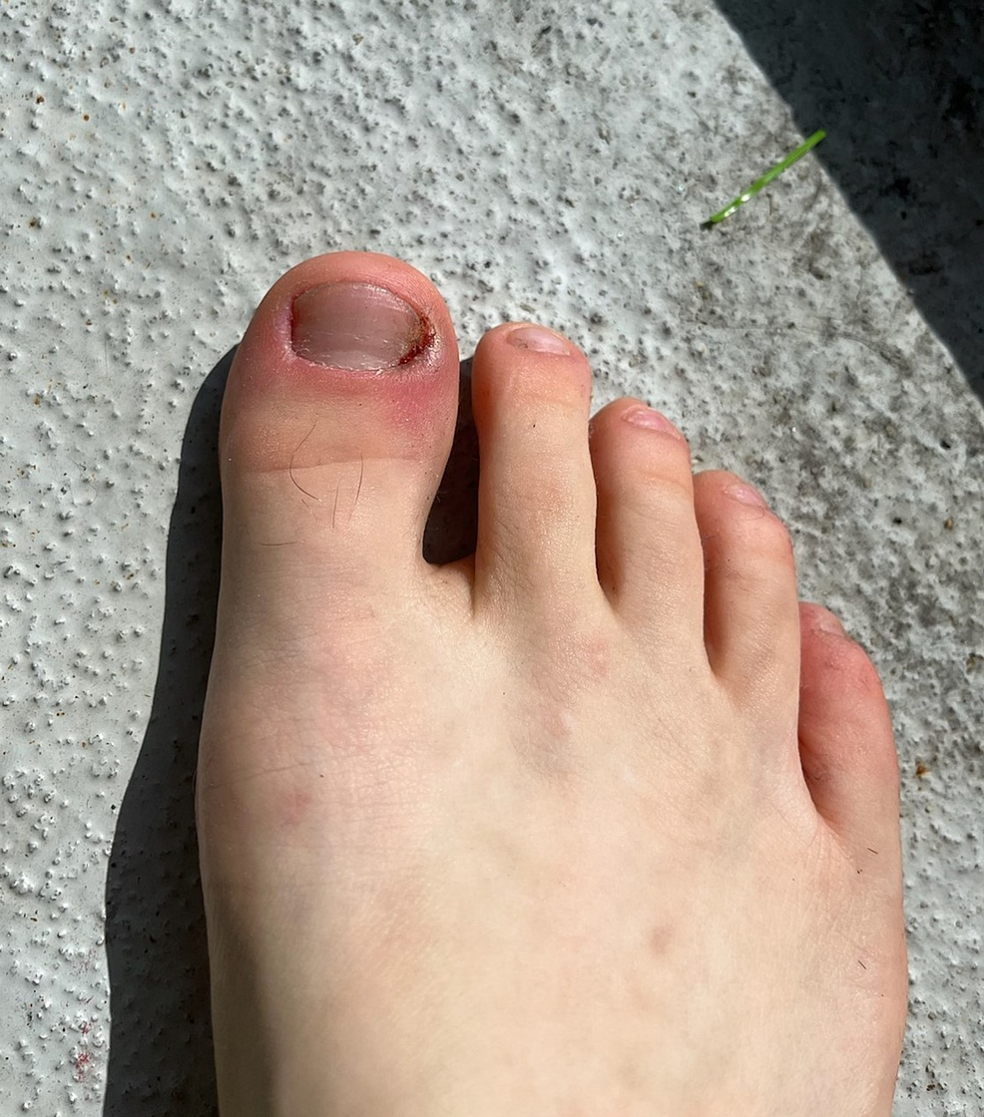
Four-week survey of the right foot. Paronychia is still noted on the lateral border of the first toenail. A slight improvement was noted in the overall nail length and extent of damage, particularly on the medial side of the first toenail.

His foot did show a partial resolution of the infection, as well as medial nail growth (Figure 5). However, he continued to endorse a constant feeling of needing to pick and shape his nails.

He was further given addiction management and psychiatric resources, as well as more topical antibiotics in the case of any digital infection. Additionally, he was connected to counseling and therapy resources specifically geared toward lower-income and unsheltered patients.

## Discussion

This 32-year-old unsheltered homeless male with a year-long deterioration of his physical and mental health developed onychotillomania as a result. Self-injurious behavior has been noted to be a relatively common phenomenon among younger, unsheltered males [11]. However, there have not been specifically described cases of onychotillomania in this high-risk patient group. Based on searches in multiple medical databases and to the best of our knowledge, our patient represents the first documented case of this condition among an unsheltered homeless patient. The progression of his disease was likely multifaceted [12]. A combination of personal stressors such as a traumatic family death, financial struggles, and substance use could have been the inciting factors for the eventual development of onychotillomania.

As a condition, onychotillomania typically occurs in conjunction with other psychiatric diagnoses. There have not been any observational or controlled studies conducted to evaluate associations of onychotillomania among psychiatric disorders. However, most patients diagnosed with onychotillomania have psychiatric comorbidities, specifically depression, anxiety disorders, and obsessive-compulsive disorder [12]. In a review by Halteh et al., whereby 21 cases reported in the literature were evaluated, six cases were associated with depression, including persistent depressive disorder and major depression with or without psychosis. Two cases were associated with specific phobias. One case was associated with presumptive psychosis and one with a hypochondriacal delusion of nail disease [1,13]. A total of nine reported cases did not include a psychiatric assessment, and only one case did not have any diagnosable psychiatric comorbidity. Although, thus far, OCD has not been diagnosed in cases of onychotillomania, patients have described tension before picking nails, followed by relief after the behavior, potentially pointing to an OCD-like behavior [14,15].

Our patient’s compromised mental health likely had a strong association with the development of his nail-picking. He did not have any history of suicidal ideations or attempts in the past. The overall timeline and severity of his symptoms mean that he had likely been experiencing symptoms of suboptimal mental health for at least six months. Additionally, although a comprehensive mental health assessment was not possible, he had symptoms indicative of major depressive disorder such as anhedonia, hopelessness, insomnia, and excessive guilt. It is difficult to exactly ascertain how much of his condition is the long-term development of an adjustment disorder brought about by his substance use and eviction.

Intervention by a multidisciplinary team of psychiatrists and dermatologists may have led to a subjective improvement in his symptoms, but four weeks later, his overall fingernail health was roughly the same, with a slight improvement noted in the toenails. This is also due to the lack of any standardized, evidence-based treatment [15]. As a result, much of the treatment is dispensed on a case-by-case basis. Certain pharmacologic treatments such as topical steroids can help in achieving clinical success with respect to reversing nail changes [16]. However, our patient’s lack of health insurance, decreased ability to attend follow-up appointments, aversion to medications, and lack of appropriate storage for medications meant that pharmacologic therapy was not optimal for this case. Our patient described the tactile stimulation toys as being the most helpful in dealing with the urge to pick his nails. It is possible that the manipulation of the clay may provide similar feelings of relief as the nail-picking behaviors.

The concurrent presence of substance use disorders likely confounded the treatment of our patient’s condition. This is because methamphetamine usage is generally associated with an increased risk of delusions of parasitosis and chronic skin-picking behavior [17]. However, our patient denied any ideas of parasitosis, and his picking behavior was mainly limited to the fingernails and toenails, with sporadic excoriations of other body parts. It would be expected for methamphetamine-induced excoriations to be present more diffusely on the trunk, face, and extremities. Dermatillomania is a nonspecific term for excoriation disorder (or chronic skin-picking), which also manifests on other body parts [18]. Although it can also be localized to the area surrounding the nails, our patient’s excessive fixation on the nail itself meant that the trauma to the periungual skin was likely an extension of the onychotillomania.

The most important aspect of our patient’s story is how the lack of shelter led to the significant worsening of his overall health. More research is required on the exact effects of trauma resulting from unsheltered homelessness, such as associations with depression, anxiety, or self-harm behaviors. The effects of current socioeconomic hardships will likely be felt over a prolonged period of time, but such patterns with younger patients becoming homeless may represent an alarming public health emergency.

## Conclusions

Onychotillomania is a rare psychodermatosis that involves repetitive picking, biting, or pulling of the fingernails and toenails and sometimes includes the periungual skin. The Diagnostic and Statistical Manual-5 (DSM-V) does not include onychotillomania as a separate diagnosis, but it is generally categorized under the body-focused repetitive behavior disorder, which is under the larger umbrella of obsessive-compulsive and related disorders. The pathophysiology of this condition is poorly understood, but some patients have been observed to have momentarily psychological relief by the repetitive act of picking. Complications of this condition include permanent nail disfiguration, digital infection, and impaired daily functioning. Due to the relative rarity and multifaceted nature of this condition, there are no established evidence-based treatments, and management mostly relies on patient-specific therapeutic measures.

Our case of a 32-year-old person experiencing homelessness (PEH) was notable for the development of onychotillomania as a result of becoming unhoused. Additionally, the adequate diagnosis was initially delayed due to the presence of concurrent methamphetamine usage, which can also cause excoriation behaviors in patients. Adopting a multidisciplinary approach by providing tactile stimulation instruments and connecting to psychiatric resources led to a subjective improvement in symptoms, although overall nail health remained similar. This case represents the challenge of managing rare psychiatric-dermatologic conditions in PEH patients and highlights the need for preemptive outreach to high-risk populations.
